# Use of Monocyte-Derived Macrophage Culture Increases Zika Virus Isolation Rate from Human Plasma

**DOI:** 10.3390/v11111058

**Published:** 2019-11-14

**Authors:** Emilia Sippert, Bruno C. Rocha, Felipe L. Assis, Suzan Ok, Maria Rios

**Affiliations:** Laboratory of Emerging Pathogens (LEP), Division of Emerging Transfusion Transmitted diseases (DETTD), Office of Blood Research and Review (OBRR), Center for Biologics Evaluation and Research (CBER), Food and Drug Administration (FDA), Silver Spring, MD 20993, USA; Emilia.Sippert@fda.hhs.gov (E.S.); brunocr2@gmail.com (B.C.R.); Felipe.Assis@fda.hhs.gov (F.L.A.); suzan.ok@gmail.com (S.O.)

**Keywords:** Zika virus, cell culture, primary isolation, in vitro model, infection

## Abstract

Viral isolation is desirable for many reasons, including development of diagnostic assays and reference materials, and for virology basic research. Zika virus (ZIKV) isolation from clinical samples is challenging, but isolates are known to infect various cell lines. Here, we evaluated suitability of Vero, C6/36 and JEG-3 as host cells, for direct isolation of ZIKV from human plasma. We also assessed the use of primary monocyte-derived macrophages (MDMs) culture to enhance ZIKV isolation from human plasma samples followed by virus expansion in Vero, C6/36 and JEG-3 cultures. Direct inoculation of cell lines with 42 ZIKV-RNA positive samples resulted in isolation rates of 9.52% (4/42) in Vero and C6/36, and of 7.14% (3/42) in JEG-3 cells. Inoculation of plasma in MDMs followed by supernatant testing by TaqMan RT-PCR, resulted in 33/42 (78.57%) ZIKV-RNA-positive supernatants, which expansion in cell lines increased isolation rates to 24.24% (8/33) in Vero and to 27.27% (9/33) in C6/36 and JEG-3 regardless of the presence of ZIKV-antibody. Isolates generated in JEG-3 cells were also produced in Vero and C6/36 with similar viral titers. These results suggest that efficiency of ZIKV isolation from human plasma can be enhanced when MDM culture is used before viral expansion in cell lines.

## 1. Introduction

Zika virus (ZIKV) is an emerging mosquito-borne virus from the genus *Flavivirus*, which also includes West Nile virus (WNV), dengue virus (DENV), yellow fever virus and others. ZIKV was first isolated in 1947 from a sentinel macaque in Uganda’s Zika forest [1]. Reports of human infection with ZIKV were scarce until 2007 when a sizable outbreak occurred in the Yap Island in Micronesia [2,3], which was followed by a large outbreak of ZIKV in French Polynesia [4] and other regions of the Pacific in 2013/2014. In 2015, ZIKV was first reported in Brazil and spread to many countries in South, Central, North America and the Caribbean [5]. In 2016, ZIKV gained global attention when the association of ZIKV with congenital microcephaly, severe fetal brain abnormalities and Guillain-Barré syndrome were reported [6] leading WHO to declare ZIKV a Public Health Emergency of International Concern [7]. Since then, a great effort has been made to understand several ZIKV aspects, such as its biology, epidemiology, pathogenesis and virus-host interactions.

ZIKV is spread mostly by the bite of infected *Aedes aegypti* (*Ae. aegypti*) and *Ae. albopictus* mosquitoes. However, alternative transmission routes have been identified, such as pregnancy [8,9], sexual contact [10,11] and blood transfusion [12,13]. In addition, infectious ZIKV particles have been found in saliva, urine, semen and breastmilk [14,15,16]. Most ZIKV infections (80%) show no signs or symptoms while the virus circulates in the blood, raising great concern about blood transfusion safety [2]. To minimize the risk of ZIKV transmission through transfusion, blood donations are currently screened by nucleic acid tests (NAT) for ZIKV-RNA [17]. 

Although the detection of ZIKV-RNA allows for the prevention of its entry in the blood supply to ensure blood safety, it may also produce false positive results. Therefore, in clinical settings, virus isolation is used to confirm infection prior to seroconversion, which is important for pregnancy management. In addition, viral isolation from human samples enables several other studies related to the biology of infection and genetic variability.

Primary isolation and propagation of ZIKV, like other flaviviruses (DENV and WNV), have been performed using cell lines such as Vero (kidney fibroblasts from African green monkeys) and C6/36 (cells from *Ae. albopictus* larvae); however, unlike these other flaviviruses, ZIKV isolation has been challenging using these cell culture systems [18]. Susceptibility of other cell culture systems to infection with existing isolates of ZIKV strains has already been reported [19,20,21,22]. However, there are no studies comparing the efficiency of different cell culture systems for ZIKV isolation directly from the clinical specimens. Since ZIKV has been described to infect and replicate in blood monocytes and primary monocyte-derived macrophages (MDM) [23,24,25], Vero, C6/36 and human placenta (JEG-3) cell lines [19], we set out to compare efficiency of infectivity using those cell lines and to investigate whether infecting MDM with plasma specimens before infecting cell lines (Vero, C6/36 and JEG-3) would increase the likelihood of rescuing ZIKV from human samples. Our goal was to identify which cell culture system would increase the chance of rescuing ZIKV from plasma specimens and generate a high yield of virus for future experiments.

## 2. Materials and Methods 

### 2.1. Specimens

This study included 42 plasma samples collected from blood donors during the 2016 outbreak in Puerto Rico and Florida that tested reactive for ZIKV-RNA by NAT assay. The samples had ZIKV-RNA titers ranging from 8.0 × 10^1^ to 2.5 × 10^10^ copies/mL, and 36 samples had more than 1 × 10^3^ copies/mL. These specimens had also been tested for IgG and IgM. Study protocols used in this research were reviewed and approved by the FDA Research Involving Human Subjects Committee, protocols #17-001B and #03-120B. 

### 2.2. Infectivity Studies 

#### 2.2.1. In Vero, C6/36 and JEG-3 Cell Line Culture Systems

Vero (WHO stock), C6/36 (ATCC # CRL-1660) and JEG-3 (ATCC # HTB-36) cells were maintained in Minimum Essential Medium Eagle (MEM) (Gibco-BRL, Gaithersburg, MD) supplemented with fetal bovine serum (FBS) (Hyclone, Logan, UT; respectively at 5, 5 and 10%), penicillin (100 IU/mL) and streptomycin (100 μg/mL) (Gibco BRL) at 37 °C (Vero and JEG-3 cells) or at 30 °C (C6/36) in a humidified atmosphere containing 5% CO_2_.

Virus isolation was performed by infecting Vero, C6/36 and JEG-3 cells in 6-well plates at 80% confluence with 20 µL/cm^2^ of plasma diluted 1:2 in MEM containing 2% FBS for 1 h with gentle rocking every 15 min, followed by addition of fresh medium and incubation under the conditions described above. The cells were observed daily for cytopathic effect (CPE) as an indicator of infectivity. Supernatants were harvested when extensive CPE was observed, or at Day 6 post infection, if no CPE was observed. Two additional passages (P2 and P3) of all samples were performed in 6-well plates using 20 µL/cm^2^ of supernatant from the first culture (P1). ZIKV-RNA titers in the supernatants from passage 3 (P3) were determined by TaqMan reverse-transcriptase PCR (RT-PCR), and infectious particle titers were determined by Focus Forming Assay (FFA).

#### 2.2.2. In Monocyte-Derived Macrophages (MDM) Primary Cell Culture System

**Figure 1:**
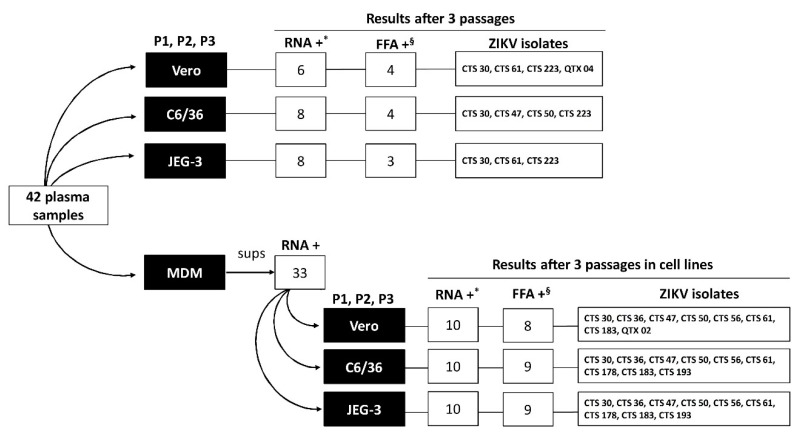
Schematic representation of the results obtained using different cell culture system for Zika virus (ZIKV) isolation from 42 plasma samples. *Number of ZIKV-RNA positive samples in P3 supernatants of Vero, JEG-3 and C6/36 cells. ^§^ Number of samples that generated ZIKV isolates. Sups: supernatants.

Elutriated human peripheral blood monocytes were kindly provided by the National Institutes of Health Blood Bank (Protocol #03-120B) for culture setting as previously described [26]. Briefly, monocytes were plated in T12.5 flasks at concentration of 5 × 10^6^ cells/flask in RPMI 1640 (Gibco-BRL) supplemented with penicillin (100 IU/mL) and streptomycin (100 μg/mL), 10% FBS and 25 ng/mL macrophage colony-stimulating factor (M-CSF) (PeproTech, Rocky Hill, NJ). The cell culture was incubated at 37 °C with 5% CO_2_ to allow differentiation into MDM. After 7 days of culture, the MDM cells were used to perform ZIKV isolation by adding 20 µL/cm^2^ of ZIKV-RNA reactive plasma diluted 1:2 in RPMI 1640 containing 2% FBS and incubating for 1 h at 37 °C with gentle rocking every 15 min. Then, fresh medium was added to the flasks and cultures incubated at 37 °C with 5% CO_2_. Culture supernatants were collected at day 7 post infection and tested for ZIKV-RNA by TaqMan RT-PCR. ZIKV-RNA positive MDM-supernatants were inoculated into Vero, JEG-3 and C6/36 cell cultures for 3 consecutive passages in each corresponding cell line (Figure 1) and tested by TaqMan RT-PCR and FFA as described above. 

### 2.3. ZIKV TaqMan RT-PCR

Total RNA was extracted from 140 µL of culture supernatant using the QIAamp Viral RNA Mini Kit (Qiagen, Valencia, CA) in accordance with the manufacturer’s instructions. Nucleic acid was eluted in 60 µL and 10 µL used per reaction. Samples were tested for ZIKV by TaqMan RT-PCR using primers and probes previously published by Lanciotti, 2008 [3]. ZIKV-RNA (PRVABC59 strain) of known copy number concentration was used as a standard for viral load determination [27].

### 2.4. Quantification of ZIKV by FFA 

FFA was performed using Vero cells for quantification of ZIKV infectious particles in P3 culture supernatants from Vero, JEG-3 and C6/36 containing more than 1 × 10^3^ copies/mL of ZIKV genome by TaqMan RT-PCR. Briefly, Vero cells were plated in 24-well plates at concentration of 1 × 10^5^/well in MEM containing 5% FBS, penicillin (100 IU/mL) and streptomycin (100 μg/mL), and kept overnight at 37 °C with 5% CO_2_. Cell monolayers at 90% confluence were infected in duplicate with tenfold dilutions of each culture supernatant. The plates were incubated for 1 h at 37 °C for viral adsorption with gentle rocking every 15 min, followed by an overlay of 1 mL of medium composed of 0.8% methylcellulose, penicillin (100 IU/mL) and streptomycin (100 μg/mL), and 2% FBS that was added to each well. After 4 days of incubation at 37 °C, the methylcellulose overlay was removed, and the wells were washed with phosphate-buffered saline (PBS) (Gibco-BRL) and then fixed with methanol (Thermo Scientific, Waltham, MA). Afterwards, 250 µL of anti-flavivirus group antigen antibody, clone D1-4G2-4-15 (Millipore Sigma, Temecula, CA) diluted 1:1000 in MEM, was added to each well, and plates were incubated for 1 hour with gentle agitation at room temperature. After 3 washes with PBS, plates were incubated for 1 hour with 250 µL of secondary antibody F(ab’)_2_ Fragment goat anti-mouse IgG (H+L) (Jackson Immuno, West Grove, PA) diluted 1:500 in MEM, washed 3 times with PBS and stained with 100 μL True Blue Peroxidase Substrate (Sera Care, Milford, MA). The viral titers were calculated by counting focus forming units.

## 3. Results

### 3.1. ZIKV Isolation Using Cell Lines: Vero, C6/36 and JEG-3

All 42 NAT positive plasma samples were independently inoculated in Vero, C6/36 and JEG-3 cell lines, and cultivated for 3 consecutive passages (P3). A total of 11 samples (26%) had detectable ZIKV-RNA titers in P3 culture supernatants from one or more infected cell lines (Table 1).

In Vero cells, 6 (14.29%) of the 42 samples produced detectable ZIKV-RNA titers after 3 passages following direct inoculation. These supernatants had viral loads ranging from 7.34 × 10^1^ to 2.12 × 10^9^ copies/mL, and 4 (66.67%) of them had detectable infectious viral particles in their supernatants when tested by FFA. In C6/36 cells, 8 (19.05%) of the 42 samples had positive supernatants with viral loads ranging from 4.44 × 10^1^ to 1.89 × 10^9^ copies/mL, and 4 (50%) of them had detectable infectious virus in their supernatants when tested by FFA. In JEG-3 cells, 8 (19.05%) of the 42 had positive supernatants with viral loads ranging from 4.19 × 10^1^ to 8.72 × 10^8^ copies/mL, and 3 (37.50%) of them had detectable infectious virus in their supernatants when tested by FFA (Figure 1 and Table 1). Based on the results of FFA, out of these 11 positive plasma samples, 6 ZIKV isolates contained infectious virus. Two out of 6 isolates (CTS 30 and CTS 223) were obtained from all 3 cell lines, 1/6 isolate (CTS 61) was obtained from both Vero and JEG-3 cells, 2/6 isolates (CTS 47 and CTS 50) were obtained from C6/36 cells, and 1/6 (QTX 04) isolate was obtained only from Vero cell culture. In addition, 5/11 samples were not tested by FFA due to negativity in TaqMan RT-PCR or low copy number of viral genome/mL (Figure 1 and Table 1). 

### 3.2. ZIKV Isolation Using MDM 

To account for individual susceptibility of MDM donors to viral infection [28], MDM cultures from 10 different donors were used to test the plasma samples. Supernatants from the MDM cultures inoculated with plasma were harvested on day 7 post infection and tested for ZIKV-RNA by TaqMan RT-PCR. MDM supernatants with detectable ZIKV-RNA were independently inoculated into Vero, C6/36 and JEG-3 cell cultures, and subjected to 3 subsequent passages to produce virus isolates. A total of 30 (71.42%) samples were inoculated in MDM cultures from multiple donors, varying between 2 and 7 different donors per plasma sample; and 12 (28.57%) plasmas were inoculated in MDM cultures from a single donor (Supplementary S2). Of the 42 samples, 33 (78.57%) produced supernatants positive for ZIKV-RNA by TaqMan RT-PCR with titers ranging from 6.55 × 10^1^ to 2.03 × 10^8^ copies/mL. Of these 33 positive supernatants, only 10 (30.30%) produced infectious particles after the cultivation in Vero (*n* = 8), C6/36 (*n* = 9) and/or JEG-3 (*n* = 9) (Figure 1, Table 2, Supplementary S3). Most MDM culture supernatants that produced viral isolates had ZIKV-RNA titers higher than 10^4^ copies/mL. Interestingly, we recovered an isolate from one sample (CTS 178) with 7.83 × 10^2^ copies/mL in MDM (Supplementary S3), but no isolate was recovered from 2 samples that had approximately 10^4^ ZIKV copies/mL in their MDM culture supernatants.

All 10 plasma samples that produced ZIKV isolates were inoculated in MDMs from at least 4 different donors and the number of positive supernatants varied among the samples and the MDM donors (Table 2). Of these 10 isolates produced after ZIKV rescue in MDM cultures, 7 (CTS 30, CTS 36, CTS 47, CTS 50, CTS 56, CTS 61, CTS 183) grew in all three cell lines (Vero, C6/36 and JEG-3), 2 isolates (CTS 178 and CTS 193) grew only in C6/36 and JEG-3 cells, and 1 isolate (QTX 02) was only produced in Vero cells (Figure 1, Table 2). Infectivity titers in isolates from Vero cells varied from 5 × 10^2^ to 1.4 × 10^7^ FFU/mL with a median of 3.5 × 10^5^ FFU/mL; in isolates from C6/36, ranged from 6.5 × 10^4^ to 8 × 10^7^ FFU/mL with a median of 1 × 10^7^ FFU/mL; and in isolates from JEG-3, varied from 2 × 10^4^ to 2.6 × 10^7^ FFU/mL with a median of 1.6 × 10^6^ FFU/mL (Supplementary S3).

The sample CTS 223 was inoculated in MDM cultures from 6 different donors and produced positive supernatants in all cultures (approximately 10^4^ copies/mL) but none grew in any of the cell lines to produce isolates (Table 2). However, isolate was generated when the plasma was directly inoculated in cell lines. 

### 3.3. Antibody Status Plays A Role in Viral Isolation 

Eighteen (42.86%) of the 42 samples were ZIKV antibody-negative, 19 (45.24%) were antibody-positive, and 5 (11.90%) were gray-zone (i.e., equivocal results for IgM or IgG). Using cell line culture systems, we were able to produce ZIKV isolates from 6 of the 18 (33.33%) antibody-negative sample (CTS 30, CTS 47, CTS 50, CTS 61, CTS 223 and QTX 04). No isolates were produced from direct inoculation of samples with positive or gray-zone ZIKV antibody results in any of the 3 cell lines (Table 3 and Supplementary S1). 

All 42 samples were inoculated in MDM cultures, and their supernatants were tested for ZIKV-RNA by TaqMan RT-PCR. A total of 33 of them produced RNA reactive results in one or more MDM cultures from different donors. Of these 33 MDM-supernatants with ZIKV-RNA reactive results, 17 were ZIKV antibody-negative, 12 were antibody-positive and 4 were gray-zone for IgM or IgG. Of the 17 ZIKV antibody-negative samples, 6 (33.3%) produced replicative virion that grew in Vero, C6/36 and JEG-3. Four of them (CTS 30, CTS 47, CTS 50, CTS 61) also grew when directly inoculated in cell lines while 2 of them (CTS 36, CTS 183) did not grow when directly inoculated in cell lines. 

Interestingly, the ZIKV antibody-negative sample QTX 02, which did not grow when directly inoculated in cell lines, produced replicative virion in MDM supernatant that grew in Vero cells but not in C6/36 or in JEG-3. In addition, two ZIKV antibody-negative samples (CTS 223, QTX 04) only grew when directly inoculated in cell lines but not after inoculation in multiple MDM donors for CTS 223 and single donor for QTX 04. Of the 12 ZIKV antibody-positive samples with ZIKV-RNA-positive results in MDM supernatant, only 3 (25%) (CTS 56, CTS 178 and CTS 193) produced isolates after expansion in cell lines. The same 3 samples did not produce isolates after direct inoculation in any of the 3 cell lines (Table 1, Table 2). 

## 4. Discussion

Virus isolation is essential for basic and applied research on viral structure, replication, virulence or attenuation, virus antibody interaction and evolution, as well as for molecular epidemiological studies. Viral strains are also useful for the development of diagnostic tests, reference material and vaccines. Flavivirus isolation from clinical samples has been typically performed in Vero and C6/36 cell lines with isolation rates above 50% [29,30], but ZIKV isolation has been a difficult task [18,31]. This study demonstrated a higher ZIKV isolation rate from plasma samples when using MDM culture for virus rescue followed by virus expansion in cell lines. This strategy also allowed the production of high-titer viral stocks with low passage number to be used in future experiments.

This study included plasma samples from 42 blood donors that tested reactive for ZIKV-RNA by NAT assay during the 2016 outbreak in Puerto Rico and Florida. These samples were directly inoculated in Vero, C6/36 and JEG-3 cell lines and tested after 3 passages. The recovery of ZIKV by direct inoculation in these cell lines was effective for 14.3% of the plasma samples. The frequency of ZIKV isolation in Vero and C6/36 was slightly higher compared to the rate of isolation in JEG-3 (9.52% for Vero and C6/36; 7.14% for JEG-3; Supplementary S1). In addition, all isolates produced in JEG-3 were also produced in Vero and/or C6/36 with similar concentration of infectious particles. Among the cell lines tested in this study, the highest viral titers were generally obtained from C6/36 cells either by direct inoculation with plasmas or with MDM supernatants. ZIKV titer has been reported to reach its peak at day 3 post infection in both Vero [19] and JEG-3 [32] when it starts to decrease, while in C6/36 cells, the viral titer is still increasing at day 5 post infection [19], which could explain the higher viral titers in C6/36 cells observed in our study. 

MDM cultures are reported to be susceptible to infection with ZIKV isolates [25]; however, to the best of our knowledge, there are no studies investigating whether MDM cultures would be suitable for primary isolation of ZIKV from human plasma samples. We tested 42 ZIKV-RNA positive plasma samples by inoculation in MDM cultures and found detectable ZIKV-RNA in culture supernatant of 33 (78.57%) of the 42 samples using cultures from one or more MDM donors. The ZIKV-RNA positive supernatants were inoculated in each of Vero, C6/36 and JEG-3 cell lines to produce ZIKV isolates. The results showed that isolation rates vary among cell lines from 24.24% (8/33) in Vero to 27.27% (9/33) in C6/36 and JEG-3 (Supplementary S1). Interestingly, we also observed that when MDM cultures from different donors were infected with the same plasma sample, some MDM cultures were not susceptible to ZIKV as demonstrated by the lack of detectable ZIKV-RNA in their supernatants (Supplementary S3), indicating that successful viral isolation may depend on donor susceptibility. For this reason, inoculation in MDMs from multiple donors may be needed for successful viral isolation. Of the 10 isolates produced from ZIKV-RNA positive MDM supernatants, 7 were produced in all 3 cell lines from one or more MDM donor’s supernatants, 2 of them grew in both C6/36 and JEG-3 but not in Vero cells, and 1 grew only in Vero; but all isolates produced in JEG-3 were also produced in Vero and/or C6/36 cultures, suggesting the need to use both C6/36 and Vero cell culture systems to increase the chances of virus isolation after initial inoculation of plasma in MDM cultures. In addition, MDM supernatant from samples CTS 223 and QTX 04 were TaqMan-positive, but ZIKV did not grow in any of the 3 cell lines. However, both QTX 04 and CTS 223 produced an isolate when plasma was directly inoculated into Vero cells, demonstrating the importance of Vero cells in ZIKV isolation. Conversely, inoculation of plasma in MDM culture were performed at a much later date after additional freeze/thaw, which may have impacted the efficiency of infectious particle production by MDM. 

Remarkably, 3 of 10 ZIKV isolates were produced after being rescued in MDM from ZIKV antibody-positive samples that did not infect Vero, C6/36 or JEG-3 by direct inoculation, suggesting that the antibodies in the clinical specimens were neutralizing for these cell lines, but not for MDM. It is possible that ZIKV antibodies facilitated MDM infection through interaction with cell-surface molecules such as Fcγ receptors. This phenomenon is known as antibody-dependent enhancement of viral infection and has been described for several viruses including ZIKV [33], DENV, WNV, tick-borne encephalitis virus and human immunodeficiency virus (reviewed in [34]). Therefore, rescuing the virus using MDM culture system would be a suitable approach for enhancing virus isolation from plasma samples including ZIKV antibodies. 

Our findings on ZIKV isolation are aligned with findings on other flavivirus reports for WNV and DENV. WNV isolation from plasma samples shows a higher rate when MDM is used for virus rescue followed by virus expansion in Vero cells (95%) compared to direct inoculation in Vero cells (63%) [30]. Additionally, DENV isolation and binding are compromised by high levels of anti-DENV IgM and/or IgG, which resulted in reduced virus isolation rate in C6/36 cells [29] and a decrease of DENV adsorption in Vero cells [35].

ZIKV isolation depends on a variety of factors such as viremia levels, presence of antibodies (IgM and IgG), type of clinical samples (serum, plasma, urine) and cell culture system used for isolation. Our results demonstrate MDM cultures’ suitability for ZIKV isolation from human plasma, regardless of the presence of ZIKV antibodies, with production of infectious viral particles. Thus, ZIKV isolation rates can be further increased by pre-cultivation of human plasma samples in MDM followed by expansion in cell line culture systems to allow robust production of viable virus particles. Altogether, these results suggest that circulating flavivirus-specific antibodies that may inhibit infection in cell lines, may not inhibit infection in primary MDM cultures, raising the question of whether cell lines neutralizing antibodies produced against flaviviruses are protective in vivo, and shows that antibody positive samples should not be considered as a reduced risk for flavivirus transmission to transfusion recipients.

## Figures and Tables

**Table 1:** Isolates produced from direct inoculation of human plasma in cell lines: viral load and infectivity titers in P3 supernatants.

	Antibody Test Result			Copies/mL *			FFU/mL ^†^	
Isolate	IgM	IgG	Vero	C6/36	JEG-3	Vero	C6/36	JEG-3
**CTS 30**	–	–	1.71 × 10^4^	1.67 × 10^9^	1.09 × 10^6^	1.00 × 10^1^	1.00 × 10^7^	6.00 × 10^2^
**CTS 36**	–	–	0	0	1.21 × 10^2^			
**CTS 47**	–	–	2.96 × 10^2^	1.30 × 10^9^	0		3.00 × 10^6^	
**CTS 50**	–	–	0	1.74 × 10^9^	1.55 × 10^2^		9.00 × 10^6^	
**CTS 56**	+	+	0	2.20 × 10^2^	4.19 × 10^1^			
**CTS 61**	–	–	9.58 × 10^8^	7.43 × 10^1^	6.09 × 10^5^	4.00 × 10^6^		3.00 × 10^2^
**CTS 178**	–	+	0	2.03 × 10^2^	0			
**CTS 183**	–	–	7.34 × 10^1^	0	0			
**CTS 193**	+	–	0	4.44 × 10^1^	1.05 × 10^2^			
**CTS 223**	–	–	3.44 × 10^8^	1.89 × 10^9^	8.72 × 10^8^	9.50 × 10^5^	1.70 × 10^6^	3.00 × 10^5^
**QTX 04**	–	–	2.12 × 10^9^	0	4.81 × 10^1^	7.50 × 10^6^		

Notes: (–) indicates absence and (+) indicates presence of ZIKV IgM or IgG; * ZIKV genome (copies/mL) was determined in P3 supernatants by TaqMan RT-PCR assay; ^†^ ZIKV infectious particles (FFU/mL) was determined in P3 supernatants by FFA; Blank cells: not tested by FFA.

**Table 2:** Monocyte-derived macrophages (MDMs) donor’s variability in susceptibility to infection with ZIKV-RNA positive human plasma samples as determined by expansion of virus in cell lines.

Sample ID	Antibody Test Result		MDM Positive Cultures *	Average of ZIKV (copies/mL) in MDM Cultures ^†^	FFA in Cell Lines Supernatant from P3 after Inoculated with MDM Supernatants			Average of Infectious Particle Concentration (FFU/mL) in P3 Supernatants ^§^		
	IgM	IgG			Vero	C6/36	JEG-3	Vero	C6/36	JEG-3
**CTS 30**	–	–	3/4	6.71 × 10^7^	Pos (1/3)	Pos (2/3)	Pos (2/3)	5.00 × 10^2^	8.75 × 10^6^	2.10 × 10^6^
**CTS 36**	–	–	4/7	5.35 × 10^6^	Pos (1/4)	Pos (1/4)	Pos (1/4)	4.50 × 10^5^	3.50 × 10^6^	2.10 × 10^5^
**CTS 47**	–	–	3/4	6.85 × 10^7^	Pos (2/3)	Pos (3/3)	Pos (3/3)	4.65 × 10^5^	1.37 × 10^7^	2.67 × 10^6^
**CTS 50**	–	–	3/4	7.65 × 10^7^	Pos (2/3)	Pos (2/3)	Pos (2/3)	1.43 × 10^6^	2.28 × 10^7^	2.00 × 10^6^
**CTS 61**	–	–	4/4	2.59 × 10^5^	Pos (2/4)	Pos (3/4)	Pos (3/4)	5.50 × 10^4^	2.08 × 10^7^	1.43 × 10^7^
**CTS 183**	–	–	2/4	3.10 × 10^7^	Pos (2/2)	Pos (2/2)	Pos (2/2)	1.76 × 10^5^	9.25 × 10^6^	2.05 × 10^6^
**QTX 02**	–	–	1/7	4.31 × 10^4^	Pos (1/1)	Neg	Neg	1.00 × 10^7^		
**QTX 04**	–	–	1/1	7.77 × 10^3^	Neg	Neg	Neg			
**CTS 223**	–	–	6/6	1.80 × 10^4^	Neg	Neg	Neg			
**CTS 56**	+	+	1/4	1.95 × 10^8^	Pos (1/1)	Pos (1/1)	Pos (1/1)	1.40 × 10^7^	6.00 × 10^7^	1.50 × 10^6^
**CTS 178**	–	+	4/4	2.67 × 10^7^	Neg	Pos (2/4)	Pos (1/4)		4.00 × 10^7^	1.10 × 10^5^
**CTS 193**	+	–	1/4	1.31 × 10^7^	Neg	Pos (1/4)	Pos (1/4)		1.00 × 10^7^	6.50 × 10^5^

Notes: This table represents the findings using the MDM system for 12 plasma samples, source of all ZIKV isolates produced in this study. Two (QTX 04 and CTS 223) of the 12 ZIKV isolates were not produced using MDM culture system. ( – ) indicates absence and (+) indicates presence of ZIKV IgM or IgG. *The data in this column indicates the number of positive cultures for ZIKV-RNA by TaqMan RT-PCR/total number of MDM cultures/donors tested. Pos indicates positive, Neg indicates negative by FFA. ^†^ Average of ZIKV genome (copies/mL) in positive MDM culture supernatants from day 7 post infection determined by TaqMan RT-PCR. ^§^ Average of FFU/mL calculated when more than one culture was positive (see Supplementary S3 for individual information). Blank cells: not tested by FFA.

**Table 3:** Rates of ZIKV isolation in Vero, C6/36 and JEG-3 cell lines, either by direct inoculation with plasma sample or ZIKV-positive MDM supernatant, distributed according to ZIKV antibody status.

ZIKV Antibody Status	Total	Isolation Rate Direct in Cell Lines			MDM *	Isolation Rate in Cell Lines after MDM Rescue		
		Vero	C6/36	JEG-3		Vero	C6/36	JEG-3
		Pos (%)	Pos (%)	Pos (%)	Pos (%)	Pos (%)	Pos (%)	Pos (%)
IgM‒, IgG‒	18	4 (22.22)	4 (22.22)	3 (16.66)	17 (94.44)	7 (41.18)	6 (35.29)	6 (35.29)
IgM+, IgG+	11	0 (0)	0 (0)	0 (0)	7 (63.63)	1 (14.29)	1 (14.29)	1 (14.29)
IgM‒, IgG+	5	0 (0)	0 (0)	0 (0)	3 (60)	0 (0)	1 (33.33)	1 (33.33)
IgM+, IgG‒ or IgM+, IgG Eq	3	0 (0)	0 (0)	0 (0)	2 (100)	0 (0)	1 (50)	1 (50)
IgM‒, IgG Eq or IgM Eq, IgG‒	5	0 (0)	0 (0)	0 (0)	4 (66.66)	0 (0)	0 (0)	0 (0)
	42	4 (9.52)	4 (9.52)	3 (7.14)	33 (78.57)	8 (24.24)	9 (27.27)	9 (27.27)

Notes: Eq: equivocal. * samples that were positives for ZIKV-RNA in one or more MDM supernatants. Pos indicates positive viral isolation determined by FFA.

## Supplementary Materials

The following are available online at https://www.mdpi.com/1999-4915/11/11/1058/s1. Supplementary S1. Number of ZIKV isolations according to the cell culture system used and antibody status. Supplementary S2. Number of plasma samples and MDM donors tested. Supplementary S3. Viral load and infectivity titers in MDM cultures and in P3 supernatants from cells lines inoculated with MDM supernatants.
