# Supplementary material for: Use of Monocyte-Derived Macrophage Culture Increases Zika Virus Isolation Rate from Human Plasma
Source: Viruses. 2019 Nov 14;11(11):1058. doi: 10.3390/v11111058 (PMC6893817; doi:10.3390/v11111058)
Supplement: Supplementary file 1 [file viruses-11-01058-s001.pdf]

Supplementary Materials:

Suppl. S1. Number of ZIKV isolations according to the cell culture system used and antibody status.

	Virus isolation		P-value*
	Isol. Neg. (%)	Isol. Pos. (%)	
**Direct inoculation in cell lines**			
Vero (n=42)	38 (90.48)	4 (9.52)	ref
C6/36 (n=42)	38 (90.48)	4 (9.52)	ns
JEG-3 (n=42)	39 (92.86)	3 (7.14)	ns
Antibody positive (n=19)	19 (100)	0 (0)	ref
Antibody negative (n=18)	12 (66.67)	6 (33.33)	0.008
Gray zone (n=5)	5 (100)	0 (0)	ns
**Inoculation in cell lines after MDM rescue**			
MDM followed by Vero (n=33)	25 (75.76)	8 (24.24)	ref
MDM followed by C6/36 (n=33)	24 (72.73)	9 (27.27)	ns
MDM followed by JEG-3 (n=33)	24 (72.73)	9 (27.27)	ns
Antibody positive (n=12)	9 (75)	3 (25)	ref
Antibody negative (n=17)	10 (58.82)	7 (41.18)	ns
Gray zone (n=4)	4(100)	0 (0)	ns

Isol.: isolation; Neg: negative; Pos: positive; ref: reference; * P-value calculated using Chi-square Test or Fisher Exact Test (when n<5), with significance set as α= 0.05. ns: non-statistically significant.

Suppl. S2. Number of plasma samples and MDM donors tested.

**Number of plasma samples**	**Number of MDMs donors infected**	**Number of positive samples after MDM culture ***	**Number of isolates generated †**	**Cell lines that generated isolates (number of isolates produced) §**
12	1	8/12	0/8	none
9	2	8/9	0/8	none
18	4	14/18	8/14	Vero, C6/36 and JEG-3 (6)<br>C6/36 (2)
1	6	1/1	0/1	none
2	7	2/2	2/2	Vero, C6/36 and JEG-3 (1)<br>Vero (1)
42	10	33/42	10/33	

* Number of plasma samples from which replicative virion were rescued by MDM culture regardless the number of MDM supernatants that were positives. †Number of ZIKV isolates generated in different cell lines after rescuing the virus in MDMs. §Cell lines in which the isolates were expanded after rescuing the virus in MDMs.

Suppl. S3. Viral load and infectivity titers in MDM cultures and in P3 supernatants from cells lines inoculated with MDM supernatants.

Isolate	Donors	Copies/mL*	Copies/mL*			FFU/mL†		
		M1D7	Vero	C6/36	JEG-3	Vero	C6/36	JEG-3
CTS 30	MDM 3	$1.52x10^{8}$	$8.03x10^{6}$	$5.05x10^{9}$	0	$5.00x10^{2}$	$4.50x10^{6}$	
	MDM 4	0	$1.62x10^{2}$	0	$6.54x10^{1}$			
	MDM 5	$4.40x10^{4}$	0	0	$3.40x10^{8}$			$2.00x10^{5}$
	MDM 6	$4.92x10^{7}$	0	$8.31x10^{8}$	$7.09x10^{8}$		$1.30x10^{7}$	$4.00x10^{6}$
CTS 36	MDM 3	$2.14x10^{7}$	$3.70x10^{7}$	$4.77x10^{9}$	$9.64x10^{8}$	$4.50x10^{5}$	$3.50x10^{6}$	$2.10x10^{5}$
	MDM 4	0	0	0	0			
	MDM 5	$4.81x10^{2}$	0	0	0			
	MDM 6	$5.79x10^{3}$	0	0	0	0		
	MDM 8	0	0	0	0			
	MDM 9	0	0	0	0			
	MDM 10	$1.52x10^{3}$	0	0	0	0		
CTS 47	MDM 3	$2.03x10^{8}$	$1.95x10^{7}$	$5.63x10^{7}$	$8.61x10^{8}$	$8.50x10^{5}$	$6.00x10^{6}$	$3.00x10^{5}$
	MDM 4	0	0	0	0			
	MDM 5	$1.11x10^{5}$	0	$1.03x10^{9}$	$6.47x10^{8}$		$1.60x10^{7}$	$1.70x10^{6}$
	MDM 6	$2.38x10^{6}$	$5.63x10^{7}$	$9.43x10^{8}$	$7.88x10^{8}$	$8.00x10^{4}$	$1.90x10^{7}$	$6.00x10^{6}$
CTS 50	MDM 3	$1.98x10^{8}$	$5.63x10^{7}$	$6.09x10^{9}$	$6.51x10^{8}$	$3.50x10^{5}$	$5.50x10^{6}$	$2.00x10^{6}$
	MDM 4	0	0	0	$9.79x10^{1}$			
	MDM 5	$1.69x10^{5}$	0	0	0			
	MDM 6	$3.12x10^{7}$	$9.97x10^{8}$	$4.54x10^{8}$	$7.53x10^{8}$	$2.50x10^{6}$	$4.00x10^{7}$	$2.00x10^{6}$
CTS 56	MDM 1	$1.95x10^{8}$	$6.96x10^{8}$	$6.86x10^{8}$	$6.22x10^{8}$	$1.40x10^{7}$	$6.00x10^{7}$	$1.50x10^{6}$
	MDM 8	0	0	0	0			
	MDM 9	0	0	0	0			
	MDM 10	0	0	0	0			
CTS 61	MDM 1	$6.67x10^{4}$	$2.13x10^{2}$	$7.12x10^{8}$	$1.59x10^{7}$		$6.00x10^{7}$	$2.00x10^{4}$
	MDM 8	$1.73x10^{4}$	0	0	0			
	MDM 9	$4.96x10^{5}$	$1.10x10^{8}$	$2.14x10^{9}$	$1.05x10^{9}$	$3.50x10^{4}$	$1.35x10^{6}$	$2.60x10^{7}$
	MDM 10	$4.57x10^{5}$	$4.67x10^{8}$	$1.93x10^{9}$	$9.06x10^{8}$	$7.50x10^{4}$	$9.50x10^{5}$	$1.70x10^{7}$
CTS 178	MDM 1	$1.07x10^{8}$	$1.57x10^{3}$	$6.66x10^{8}$	$3.31x10^{8}$	0	$6.50x10^{4}$	$1.10x10^{5}$
	MDM 8	$1.75x10^{2}$	0	0	0			
	MDM 9	$2.10x10^{2}$	0	0	0			
	MDM 10	$7.83x10^{2}$	0	$2.13x10^{9}$	0		$8.00x10^{7}$	
CTS 183	MDM 3	$6.12x10^{7}$	$1.84x10^{7}$	$5.19x10^{9}$	$8.10x10^{8}$	$3.50x10^{5}$	$8.50x10^{6}$	$6.00x10^{5}$
	MDM 4	0	0	$1.88x10^{2}$	$6.35x10^{2}$			
	MDM 5	0	0	0	0			
	MDM 6	$8.15x10^{5}$	$3.79x10^{5}$	$8.74x10^{8}$	$7.01x10^{8}$	$1.00x10^{3}$	$1.00x10^{7}$	$3.50x10^{6}$
CTS 193	MDM 1	$1.31x10^{7}$	0	$5.67x10^{8}$	$1.75x10^{8}$		$1.00x10^{7}$	$6.50x10^{5}$
	MDM 8	0	0	0	0			
	MDM 9	0	0	0	0			
	MDM 10	0	0	0	0			
QTX 02	MDM 3	$4.31x10^{4}$	$3.64x10^{9}$	0	0	$1.00x10^{7}$		
	MDM 4	0	0	0	0			
	MDM 5	0	0	0	0			
	MDM 6	0	0	0	0			
	MDM 8	0	0	0	0			
	MDM 9	0	0	0	0			
	MDM 10	0	0	0	0			

M1D7: culture supernatant was collected at Day 7 post-infection and tested by TaqMan RT-PCR assay. *ZIKV genome (copies/mL) was determined by TaqMan RT-PCR assay; † The number of ZIKV infectious particles (FFU/mL) was determined by FFA. Blank cells: not tested by FFA.
